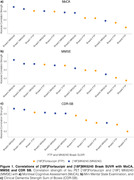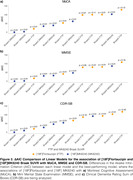# Direct comparisons of the associations between the tau PET tracers [^18^F]Flortaucipir and [^18^F]MK6240 with tests of general cognitive performance ‐ The HEAD study

**DOI:** 10.1002/alz.091693

**Published:** 2025-01-09

**Authors:** Livia Amaral, Guilherme Povala, Bruna Bellaver, Guilherme Bauer‐Negrini, Firoza Z Lussier, Pamela C.L. Ferreira, Juli Cehula, Dana Tudorascu, William J. Jagust, William E Klunk, Val J. Lowe, Hwamee Oh, David N. soleimani‐meigooni, Belen Pascual, Brian A. Gordon, Pedro Rosa‐Neto, Suzanne L. Baker, Tharick A. Pascoal

**Affiliations:** ^1^ University of Pittsburgh, Pittsburgh, PA USA; ^2^ Lawrence Berkeley National Laboratory, Berkeley, CA USA; ^3^ Department of Radiology, Mayo Clinic, Rochester, MN USA; ^4^ Brown University, Providence, RI USA; ^5^ Memory and Aging Center, Weill Institute for Neurosciences, University of California, San Francisco, San Francisco, CA USA; ^6^ Houston Methodist Research Institute, Houston, TX USA; ^7^ Washington University in St. Louis School of Medicine, St. Louis, MO USA; ^8^ Translational Neuroimaging Laboratory, The McGill University Research Centre for Studies in Aging, Montréal, QC Canada

## Abstract

**Background:**

Tau PET tracers are employed to measure the accumulation of tau in vivo in the brain. Each tau tracer possesses unique characteristics, including binding affinity, sensitivity, and specificity to tau aggregates. This study leverages the HEAD study dataset, which is currently performing baseline tau PET tracers and conducting multiple clinical and cognitive assessments. The objective of this study is to elucidate the relationship between tau PET tracers [^18^F]Flortaucipir and [^18^F]MK6240 and commonly used tests of general cognitive performance.

**Methods:**

We assessed 170 individuals (107 cognitively unimpaired, and 63 cognitively impaired) with available PET [^18^F]Flortaucipir [^18^F]MK6240 and cognitive assessments (MoCA, MMSE, and CDR‐SB). We calculated tau PET SUVR for [^18^F]Flortaucipir and [^18^F]MK6240 using the inferior cerebellar grey matter as a reference. Pearson correlations were performed between SUVR values for each Braak region and MoCA, MMSE, and CDR‐SB. In addition, linear regression models accounting for age, sex, years of education, HEAD site, and diagnosis were used to estimate the relationships. The Akaike Information Criterion (AIC) was utilized to evaluate the models, with smaller AIC values signifying better performance.

**Results:**

Person correlation coefficients (r), which were calculated without considering any covariates, demonstrated higher numerical values between [^18^F]MK6240 and the scores of MoCA, MMSE, and CDR‐SB across the Braak regions (Figure 1). Similarly, when we performed linear regressions that accounted for relevant covariates, we found that the [^18^F]MK6240 SUVR in Braak 1 and Braak 4 regions exhibited a stronger association with cognitive tests than in other Braak regions or any region with [^18^F]Flortaucipir SUVR (Figure 2).

**Conclusions:**

Both [^18^F]Flortaucipir and [^18^F]MK6240 SUVR demonstrated a robust association with tests of general cognitive performance commonly used in AD research and clinical practice. Generally, Braak regions 1 and 4 exhibited a stronger association with general cognitive performance using all tests, whereas Braak regions 5‐6 showed the weakest association.